# eConsults for Interventional Endoscopy Improve Clinician Satisfaction

**DOI:** 10.1055/a-2889-0347

**Published:** 2026-06-25

**Authors:** Michael T. O’Brien, Anthony M. Gamboa, Mark J. Radlinski, Sara N. Horst, Patrick S. Yachimski

**Affiliations:** 1 Department of Medicine 12328 Vanderbilt University Medical CenterNashvilleTennesseeUnited States; 2 Department of Medicine, Division of Gastroenterology 12328 Vanderbilt University Medical CenterNashvilleTennesseeUnited States

**Keywords:** endoscopic ultrasonography, pancreatobiliary (ERCP/PTCD), ERC topics, tissue diagnosis

## Abstract

**Background and Aims** As part of an effort to streamline our institutional process for referral and scheduling of interventional endoscopy procedures; facilitate ease of referral for these procedures; and standardize documentation of critical steps in the chain of referral, review, and scheduling, we devised and implemented an eConsult service for interventional endoscopy and analyzed its performance by uptake and provider satisfaction.

**Methods** An eConsult electronic order template was designed and implemented for outpatient referrals for interventional endoscopic procedures. Following 12 months of operation of this eConsult platform, referring providers were asked to rate their satisfaction with eConsult referrals.

**Results** In all, 129 eConsults were completed within a 12-month period, with steady monthly growth throughout. About 89.5% of referring providers indicated that “eConsult has made it easier for me to refer patients for endoscopic retrograde cholangiopancreatography/endoscopic ultrasound,” while 84% indicated “I would like to see eConsult expanded to other service lines.”

**Conclusion** eConsults appear to be an efficient and effective mechanism for referral, clinically documented review, and scheduling of outpatient interventional endoscopy procedures.

## Introduction

Open access endoscopy is the practice of referral for outpatient endoscopic procedure(s) without prior clinic consultation with the gastrointestinal endoscopist. Appropriate use of open access endoscopy assumes that the ordering provider understands the indication(s) for the requested endoscopic procedure(s), discusses the indication(s) for the procedure(s) with the patient prior to referral, and provides relevant medical records with referral to the gastrointestinal endoscopist. 1 In many instances, the endoscopist may not review these records prior to the day of the procedure.

Open access endoscopy is typically most appropriate for endoscopic procedures with commonly accepted indication(s), often diagnostic in nature, in relatively healthy and fit patients. The classic example of this would be referral for colonoscopy for colorectal cancer screening. In such instances, a prior clinic consultation with the endoscopist is deemed unnecessary, is perhaps wasteful of medical resources, and potentially risks delay in expedient procedure scheduling.

While current practice guidelines do not prohibit open access endoscopy for interventional endoscopic procedures, 1 referral for endoscopic retrograde cholangiopancreatography (ERCP) and endoscopic ultrasound (EUS), for instance, is often more nuanced and complex for a number of reasons. Referring providers may be less familiar with the appropriate use of ERCP and EUS and may refer patients for procedures with questionable or no clinical indication. The risk profile for ERCP and some EUS, including the rapidly evolving field of interventional EUS, is higher than for diagnostic upper gastrointestinal endoscopy and colonoscopy, and patients may be unprepared for an informed consent discussion without prior consultation with the gastrointestinal endoscopist. Patients requiring interventional endoscopic procedures may be of advanced age or have coexistent medical illness, which may influence the risk/benefit calculus of the procedure.

Long-standing practice among the interventional endoscopy service at our institution has been to carefully vet referrals for interventional procedures. All outpatient procedure referrals for ERCP, EUS, or other interventional procedures are forwarded for review to one of the interventional endoscopists. Upon review of relevant available records, the endoscopist may then elect to either approve scheduling of the procedure, suggest an alternative such as formal clinic consultation or further ancillary testing, or communicate directly with the referring provider for clarification of indication(s) or to discuss the clinical issue at hand. The rationale for this approach is to avoid or eliminate scheduling of inappropriate procedures without prior physician review. The downsides of this approach include nonreimbursed time and effort spent reviewing records and the potential for delays in scheduling, the latter particularly if relevant records or radiologic images are not readily available for review.

As part of an effort to further streamline our institutional process for referral and scheduling of interventional endoscopy procedures; facilitate ease of referral for these procedures; and standardize documentation of critical steps in the chain of referral, review, and scheduling, we devised and implemented an eConsult service for interventional endoscopy. eConsults have emerged as an efficient, virtual service option for selected clinical services. This brief report describes the implementation and initial 12-month utilization of this service and reports acceptance/satisfaction assessment of the eConsult service.

## Methods

This study was approved by the Institutional Review Board at Vanderbilt University Medical Center (IRB #260045) and data were obtained via the electronic medical record. Patient consent was not required per IRB/VUMC guidelines.

The first phase of this project consisted in design and rollout of an “eConsult to interventional endoscopy” template, accessible in provider order entry in the electronic medical record (EPIC, Verona, Wisconsin United States) at Vanderbilt University Medical Center (VUMC). In the order template, the referring provider or delegate was prompted to enter the procedure being requested (ERCP, EUS, or other) and given the option to free text clinical information regarding the request. The template prompted the referring provider to mark the request for review by a specific interventional endoscopist or any/first available. Documentation of patient consent to eConsult, with verbal consent sufficient for this purpose, was required in the order template. This template was available via electronic order entry to all providers within VUMC.

Electronically signed “eConsult to interventional endoscopy” orders were routed to an in-basket pool consisting of three interventional endoscopy faculty, one of whom would review the request, review relevant clinical data and radiologic images available in the electronic medical record, and then submit a case request to endoscopy scheduling if the requested procedure was deemed appropriate. The endoscopist was also required to assign a Current Procedural Terminology (CPT) code to the eConsult service based on time spent completing the eConsult and whether additional electronic or verbal communication with the referring provider was required in order to complete the eConsult (for instance, CPT code 99451 for 5–10 min time spent, with no additional communication with the referring provider). Once electronically signed, the eConsult note was automatically routed to the electronic in-basket of the ordering provider for closed loop communication.

Monthly volume of eConsults requested/completed was tabulated for an initial 12-month period following rollout. Following 12 months, the second phase of this study consisted of an electronic survey (REDCap, Vanderbilt University Medical Center, Nashville, TN, United States), consisting of two statements requiring yes/no response: (1) “eConsult has made it easier for me to refer patients for ERCP/EUS” and (2) “I would like to see eConsult expanded to other service lines.”

## Results

The majority of eConsult requests originated from one of five subspecialty groups: general gastroenterology, hepatology, hepatobiliary surgery, medical oncology, and surgical oncology. Nearly all requests were for ERCP or EUS or both, with the remainder for endoluminal stent, endoscopic mucosal resection, or Barrett’s endotherapy.

**Fig. 1:**
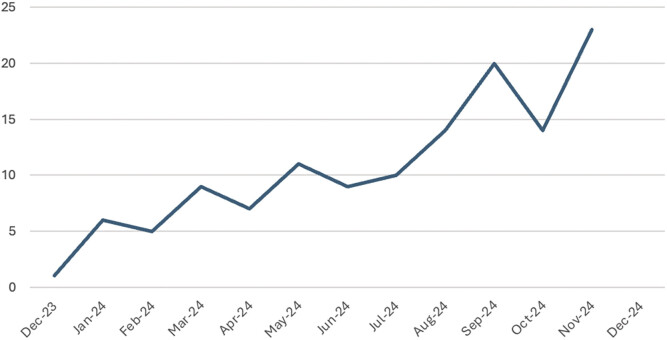
eConsult volume by month.

A total of 129 eConsults were completed within the initial 12 months following rollout, with steady growth from 1 eConsult in the first month (December 2023) to 23 eConsults in the twelfth month (November 2024) ( Fig. 1 ). Completion of an eConsult resulted in a billable service for the endoscopist completing the eConsult in 71% (92/129) of referrals. In 24 instances, a billable service was not permissible if an additional billable service had been performed by another provider in the gastroenterology practice within 14 days of the eConsult encounter.

At the completion of the initial 12-month period, 52 referring providers who had placed eConsult orders were invited to complete a satisfaction survey. The survey response rate was 37% (19/52). In total, 89.5% (17/19) of respondents indicated that “eConsult has made it easier for me to refer patients for ERCP/EUS,” while 84% (16/19) indicated “I would like to see eConsult expanded to other service lines.”

## Discussion

This manuscript reports successful design and implementation of an eConsult system for outpatient interventional endoscopy procedure referrals. Prior studies suggest that eConsults for gastroenterology can reduce unnecessary clinic visits and improve access to care, 2 but limited data exist for use of eConsults for access to interventional endoscopy services. Advantages of this eConsult system include a brief and standardized order entry template conducive to time-sensitive completion by the referring provider or delegate, clear documentation of task completion and clinical decision-making in the electronic medical record, and automated charge capture for clinical service provided. Growth in volume of referrals proved robust within the first 12 months of implementation and the eConsult service garnered high satisfaction scores.

Billing requirements and revenue generation from eConsults are early and evolving in implementation. This study showed successful revenue and productivity capture for clinicians who often are assisting in complex decision-making. Irrespective of whether criteria for a billable service were met, the endoscopist completing the eConsult received a Relative Value Unit (RVU) credit of 0.5 RVU (for a CPT code of 99451, the minimum allowable service)—this credits effort for previously uncompensated work the endoscopist had already been performing in reviewing referrals prior to implementation of the eConsult mechanism.

Future investigation may be desirable to assess whether an eConsult mechanism for interventional endoscopy reduces patient wait times, an effect which has been identified in other specialties. 3 4 This was not an assessable metric in our practice, as an explicit and longstanding goal has been to review and approve scheduling requests for outpatient ERCP/EUS referrals within 48 h of receipt both before and after inception of the eConsult platform. Additionally, further research could delineate how the eConsult mechanism affects patient outcomes, such as patient satisfaction or rates of adverse events (e.g., pancreatitis), compared to a model where patients are seen in advanced endoscopy clinic first. It is also worth noting that most of the referrals came from specialties already very familiar with advanced endoscopy, and referrals from other providers may demonstrate differing levels of complexity. Even in this situation, an eConsult is a useful framework to help clinicians understand next steps for patient care. Our survey response rate of 37% was a bit low but contained little negative feedback, indicating a useful framework. The clinic capacity in this timeframe for in-person visits did not decrease, indicating appropriate use for eConsults compared to in person evaluations. A final limitation is that we did not have access to the total number of interventional endoscopy consults in this time frame, which would have been a useful comparison with the number of eConsults.

In conclusion, our survey response data showing improved ease of advanced endoscopy consultation and desire to expand eConsults to other service lines suggest that clinicians were satisfied with this tool and its ability to improve patient care. With regard to expansion to other service lines, we have recently expanded the availability of eConsults to patients with incidentally detected pancreatic cysts. This is a common referral diagnosis to pancreatobiliary clinic, and in many instances a recommendation regarding ongoing surveillance can be made to the referring provider and patient without formal office consultation. Additional future steps include a goal to expand eConsult order requests to referring providers outside our institution, although implementation of this may require a shared common electronic medical record platform.
